# Feasibility of trancutaneous auricular vagus nerve stimulation in Black and Hispanic/Latino people with peripheral neuropathy

**DOI:** 10.3389/fpain.2024.1516196

**Published:** 2025-01-17

**Authors:** Marlon L. Wong, Eva Widerström-Noga, Jessica L. Bolanos, Gabriel Gonzalez, Frank J. Penedo, Peter J. Hosein, Melissa M. Tovin, Juan P. Gonzalez, Lisa M. McTeague

**Affiliations:** ^1^ Department of Physical Therapy, Miller School of Medicine, University of Miami, Miami, FL, United States; ^2^ The Miami Project to Cure Paralysis, University of Miami, Miami, FL, United States; ^3^ Sylvester Comprehensive Cancer Center, Miller School of Medicine, University of Miami, Miami, FL, United States; ^4^ Department of Psychiatry and Behavioral Sciences, Medical University of South Carolina, Charleston, SC, United States

**Keywords:** transcutaneous auricular vagus nerve stimulation (taVNS), transcutaneous auricular nerve stimulation, noninvasive vagus nerve stimulation, peripheral neuropathy, chemotherapy-induced peripheral neuropathy, health disparities, feasibility

## Abstract

**Introduction:**

Peripheral neuropathy (PN) is the most common neurodegenerative disorder, and the primary causes are chemotherapy-induced peripheral neuropathy (CIPN) and diabetic neuropathy (DN). Transcutaneous auricular vagus nerve stimulation (taVNS) is a promising non-pharmacological and non-invasive intervention that targets key pathways involved with PN. However, research is needed to determine the feasibility, acceptability, and effects of taVNS in people with PN. It is also critical that this research on taVNS include the perspectives of Black and Hispanic/Latino patients, who are often underrepresented in research.

**Methods:**

This research was comprised of two consecutive studies: a survey and a pilot randomized sham-controlled trial (RCT). The survey assessed symptom burden, management strategies, and interest in taVNS among CIPN patients. The pilot RCT evaluated the feasibility, acceptability, and preliminary effects of taVNS in Black and Hispanic/Latino patients with CIPN or diabetic neuropathy. Participants were recruited from the University of Miami medical system, with culturally sensitive approaches to enhance minority participation.

**Results:**

The survey included 62 respondents, 78% Black or Hispanic/Latino, revealing high symptom burden and significant interest in taVNS (82% expressed moderate to high interest). The pilot RCT enrolled 28 participants, achieving a 42% recruitment rate and 86% retention. taVNS was well tolerated, with no significant adverse effects. Preliminary data indicated a decrease in neuropathic symptoms and an increased heart rate variability (HRV) during active taVNS, suggesting autonomic modulation. Tingling sensation and pain decreased by median values of 2.0 and 1.5, respectively. Additionally, the median values for standard deviation of the RR interval increased from 34.9 (CI = 21.6–44.8) at baseline to 44.8 (CI = 26.5–50.3) during intervention. Exit interviews highlighted positive participant experiences and identified potential barriers, such as protocol length and distrust in medical research.

**Conclusion:**

The findings underscore the need for novel CIPN treatments and demonstrate the feasibility of conducting taVNS research in historically underrepresented populations. High interest in taVNS and successful recruitment and retention rates suggest that culturally sensitive approaches can enhance minority participation in clinical trials. These findings will be used to develop a large clinical trial to determine the efficacy of repeated taVNS in a diverse cohort.

**Clinical Trial Registration:**

https://clinicaltrials.gov, identifier (NCT05896202).

## Introduction

Peripheral neuropathy (PN) is the most common neurodegenerative disorder, largely due increased survivorship of cancer patients treated with neurotoxic chemotherapies and the expanding diabetes epidemic (1, 2). Standard treatments for many cancers involve the use of highly neurotoxic chemotherapies; as a result, chemotherapy-induced peripheral neuropathy (CIPN) is a devastating problem, causing dysesthesias and pain for many cancer patients and survivors (3, 4). In fact, up to 90% of people who receive neurotoxic chemotherapies will develop acute CIPN and over 30% will develop chronic CIPN (5). Similarly, PN is believed to affect up to 50% of people with diabetes (DN) (6, 7). Although the precipitating factors that lead to CIPN and DN differ, both are characterized by distal symmetric dysesthesias and paresthesias in glove/stocking distributions. Further, the associated physiological mechanisms believed to underly the perpetuation of neuropathic symptoms are similar for both groups [i.e., neuroinflammation (4), autonomic dysregulation (8–10), and altered central nervous system processing] (11–13). Current treatments for PN have limited effectiveness and unfavorable risk/benefit ratios for many patients (14–17). Thus, there is a great need to investigate new treatment options for PN, and transcutaneous auricular vagus nerve stimulation (taVNS) is a promising non-pharmacological and non-invasive intervention that is worthy of investigation.

**Figure 1:**
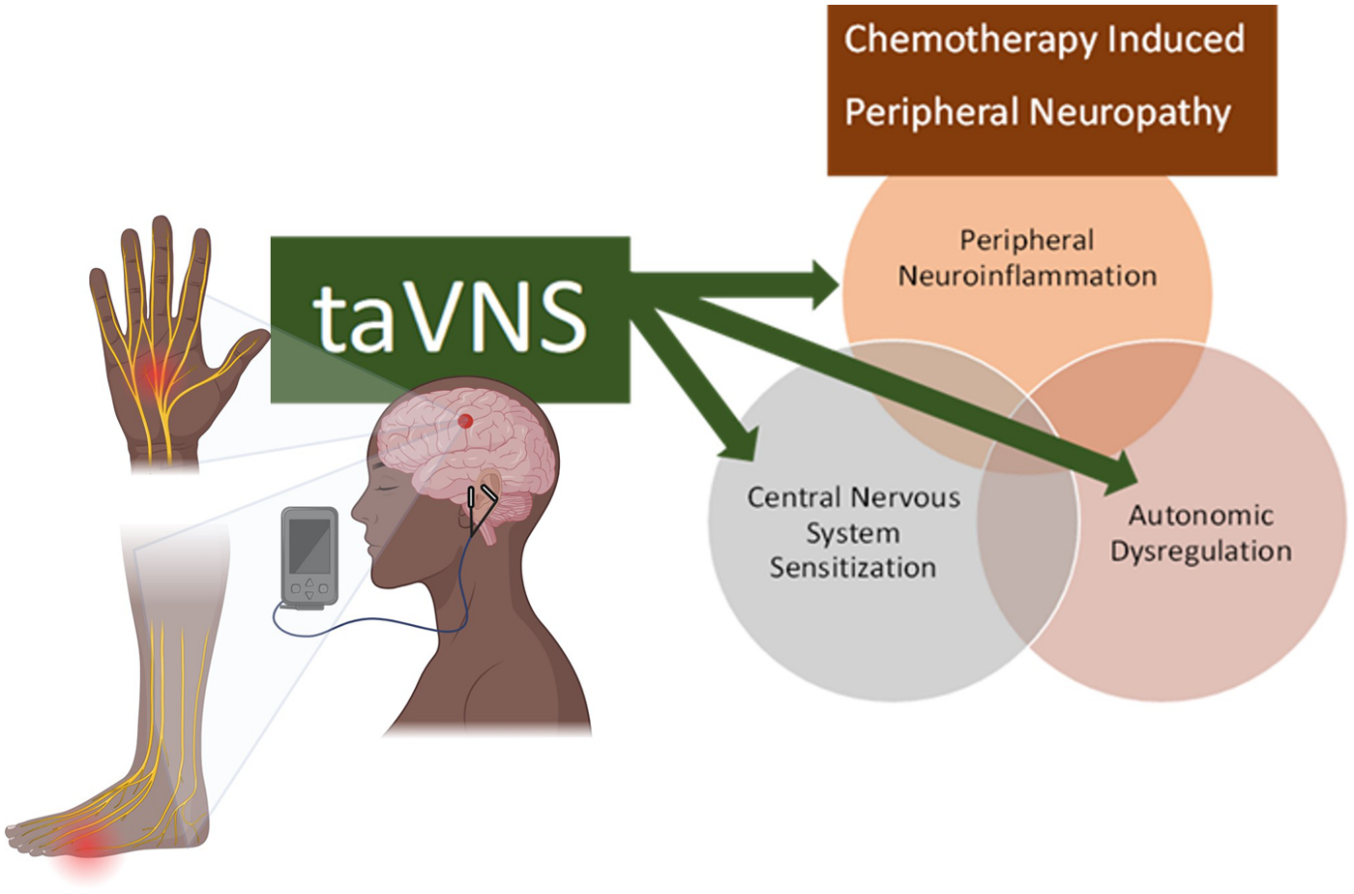
Theoretical framework for the targets of taVNS in CIPN.

Emerging evidence suggests that PN symptoms are accompanied by both structural and functional changes in the brain, namely in the pain modulation areas (18). Additionally, both CIPN and DN have been shown to be partly caused by corticospinal hyperactivity and reduced GABAergic inhibition (19–28). Thus, non-invasive brain stimulation techniques, such as taVNS, are promising tools for managing PN and warrant exploration. taVNS is particularly intriguing because it is known to act on multiple mechanisms that contribute to PN, such as dysregulation of the autonomic nervous system, inflammation (29), and structural and functional changes in the brain. The autonomic nervous system is an important regulator of stress responses, and it exhibits functional changes in PN and in other chronic pain conditions (8, 9). The vagus nerve is the primary nerve of the parasympathetic system (a key component of the autonomic nervous system), and the vagus nerve modulates pain through 3 pathways: (1) by maintaining autonomic and hypothalamic-pituitary-adrenal axis balance thereby reducing allostatic load (30), (2) via the cholinergic anti-inflammatory pathway in which action potentials in the vagus nerve inhibit production of tumor necrosis factor (TNF) and other inflammatory cytokines (31), and (3) by modulating the function of brain networks involving the cingulate cortex and insula which are involved in pain processing (32). Thus, taVNS appears to be well suited for addressing key underlying mechanisms of PN (Figure 1), and preliminary data support this potential (33, 34). Specifically, vagus nerve stimulation was found to attenuate paclitaxel-induced hyperalgesia in rats (33), and a recent RCT demonstrated significant improvement in pain and insomnia in 27 patients with CIPN after taVNS (34). Pain relief has also been observed with taVNS in other chronic pain populations including rheumatoid arthritis (35), osteoarthritis (36), and migraine (37). As a promising treatment to manage PN and improve patient outcomes, research is needed to determine the feasibility, acceptability, and effects of taVNS in people with PN. There are two priority considerations for feasibility and acceptability studies for cancer treatment. First, cancer care often places high burden on patients (38, 39) and their caregivers (40). Thus, it is important to avoid additional unnecessary or unwanted research and health care burdens for individuals with CIPN. Likewise, DN is associated with high healthcare resource utilization and costs (41). taVNS has the potential to be a relatively simple intervention that can easily be self-administered and combined with other treatments, potentially eliminating the need for costly and burdensome clinic visits. Further, it does not carry the same risks and side effects associated with many first line treatments for PN (i.e., duloxetine and gabapentin). However, research is needed to determine if there is interest in this intervention amongst this population before conducting large trials. Second, it is critical that samples accurately represent the patient population. This has proved difficult, as the cancer research, pain research, and non-invasive brain stimulation research fields are all challenged by poor racial and ethnic diversity (42, 43). It is critical that research on taVNS for PN includes perspectives of people from underrepresented communities to ensure that the research reaches all of those who may benefit from this treatment approach and to make research findings more generalizable. This is particularly important as Black patients who receive neurotoxic chemotherapies are known to be at greater risk for CIPN than non-Hispanic White people (44), and both Black and Hispanic/Latino patients are at greater risk for DN (45). The aim of this project was to assess the interest in taVNS among people with CIPN and to gain important data on the feasibility of conducting a randomized controlled pilot trial using taVNS in Black and Hispanic/Latino patients with PN.

## Materials and methods

This research consisted of 2 consecutive studies: a survey of people with CIPN, followed by a pilot randomized sham-controlled trial (RCT) of a single session of taVNS in people with CIPN or diabetic neuropathy. Although the precipitating factors that lead to chemotherapy-induced and diabetic-related neuropathies differ, the symptomology and the associated pathophysiological mechanisms believed to underly the perpetuation of neuropathic symptoms are similar (i.e., neuroinflammation, autonomic dysregulation, and altered central nervous system processing) (11–13). Thus, we included patients with diabetic neuropathy to enhance recruitment for the pilot RCT. This work is part of a larger project to develop infrastructure for a research agenda focused on equitably integrating non-invasive brain stimulation into multimodal care for peripheral neuropathy.

### Survey study

The survey study focused exclusively on CIPN. A 3-phased approach was used for recruiting survey participants. Inclusion criteria included anyone with glove or stocking distribution paresthesia or dysesthesia that developed after they received neurotoxic chemotherapies. First, the University of Miami's Consent to Contact Initiative (46) was used to identify potential participants, and these people were contacted by phone from September to December 2022. Those who indicated a desire to participate were provided with a Qualtrics survey link via email. Second, flyers were posted in oncology clinics from December 2022 to October 2023 within the University of Miami medical health care system. At the end of this period, preliminary analyses demonstrated that Black patients were underrepresented, and then an active recruiting strategy was employed targeting Black patients with CIPN within the same clinics. This active strategy involved identifying patients by medical record and then having their providers inform them during clinical visits about the survey study. The survey study also informed the recruitment strategy for the pilot RCT, and the same active strategy was utilized for recruiting Black and Hispanic/Latino patients with PN for the pilot RCT.

The survey consisted of 10 questions on current symptom burden and management strategies, 4 questions on interest in participating in nonpharmacological interventions and participating in taVNS research, 8 questions on medical history (i.e., cancer type, chemotherapies used, and symptom change after completion of chemotherapy), and 6 questions on demographics (Supplementary 1). To assess symptom burden, participants were asked to rate the average intensity of their pain, numbness, tingling, burning, and shooting/electric shocks on average within the last week using an 11-point numeric rating scale (with 0 being none and 10 being the worst imaginable), Participants also rated the change in these same symptoms since completing chemotherapy (with 0 being much worse and 10 being much better). To assess current non-pharmacological symptom management strategies used, participants were provided with a list of interventions (medication, hot or cold packs, massage, acupuncture, exercise, or electrical stimulation) and asked to select all that apply. They were also provided with the opportunity to describe other treatments that were not listed. To assess current pharmacological symptom management strategies used, participants were provided with common drugs used in this population (i.e., duloxetine, gabapentin, and opioids), with examples of medication trade names, and asked to select all that apply and given the opportunity to describe other medications that were not listed. Within the survey, participants were provided with a brief description of taVNS, how it is applied, and the proposed mechanisms of action. Then, they were asked “How interested would you be in participating in a clinical study using Vagus Nerve Stimulation to treat pain?” and provided a 5-point Likert scale ranging “not at all” to “a great deal.” This was followed up with a free text response question, “Why or why not?”

### Pilot RCT

The pilot study was a single-blinded, sham-controlled, feasibility study that was designed to examine the influence of videos, on participant expectations for pain relief, and to explore the feasibility and intended effects of taVNS in Black and Hispanic/Latino people with CIPN. Investigators were not blinded to assignment, and the effectiveness of participant blinding was assessed via questionnaire. As the primary purpose was to assess the participant expectations and feasibility, a target sample size of 24 was chosen for this study because it was estimated that this would provide the needed power to reach saturation with qualitative analysis and to assess feasibility outcomes (i.e., recruitment and retention rates).

The pilot RCT included only Black and Hispanic/Latino patients with CIPN or diabetic neuropathy. Participants were recruited from the University of Miami medical health care system from January to May 2024. Potential participants were identified by medical record and then their respective providers (i.e., oncologist or endocrinologist) informed them about the pilot study during clinical visits. Inclusion criteria included anyone with glove or stocking distribution paresthesia or dysesthesia that developed after receiving neurotoxic chemotherapies or with a diagnosis of diabetic neuropathy and who self-identified as Black or Hispanic/Latino. Exclusion criteria included (1) any unstable medical condition or medical contraindication to moderate physical exertion (e.g., unstable angina or cardiac arrythmia), (2) pregnancy, (3) presence of cognitive impairment or language barrier that impairs full autonomy in the consent process or in the ability to participate in detailed interviews, (4) implants in the head or neck, cochlear implants, or pacemaker, (5) head or neck metastasis or recent ear trauma, (6) history of seizures.

The videos tested in this RCT were developed specifically to enhance education and recruitment of participants from underrepresented communities. Participants were randomly assigned to video or control groups, and all participants completed 3 visits. The first visit consisted of ∼90 min of education on taVNS, including review of brochures and consent forms (both groups) and 3 short video segments on taVNS for the intervention group. The videos contained the same content as the brochures and consent forms, so all participants received the same information but in different formats. Further, all participants had ample opportunity to ask questions and discuss the content with the investigators. Racial and ethnic differences between participant and investigators/providers is also known to influence expectations and pain outcomes (47, 48); thus, a Black investigator provided all educational sessions for Black participants, and a Hispanic/Latino investigator provided all education sessions for Hispanic/Latino participants and in their preferred language (English or Spanish). Both investigators provided the same information to participants. At the end of the educational session, participants provided feedback on the educational materials and completed the EXPECT (49) questionnaire. The EXPECT is a 4-item questionnaire that assesses expectations for pain improvement. Each of the 4 items is scored on an 11-point scale, with 0 being no change and 10 representing complete relief (49).

For participants in the intervention group, the second visit involved an assessment battery before and after receiving 1-hour of active taVNS. The assessment battery consisted of the following:

1. Assessment of peripheral pain sensitivity using quantitative sensory testing [pressure pain threshold (Algomed, Medoc, Israel) and wind-up-ratio with pin prick (MRC Systems GmbH, Germany)]
2. Assessment of corticospinal excitability with transcranial magnetic stimulation [single pulse and paired-pulse assessments (short intercortical inhibition and intracortical facilitation using the Magpro X100, MagVenture, Alpharetta, GA)]. This data will be analyzed at a later date and presented elsewhere.
3. Examination of autonomic nervous system status using heart rate variability (HRV) assessment [via 2-lead electrocardiogram (Powerlab, AD Instruments, USA)].

Active taVNS was applied using electrodes placed at the tragus and cymba conchae, with the Soterix taVNS Stimulator, set at a frequency of 25 Hz, pulse width of 500 µs, and the stimulation intensity was set at 200% of the participants' perception threshold, as these parameters have been shown to be effective for inducing changes in heart rate in healthy participants (50).

At the end of visit 2, participants in the video treatment group received a tolerability and sham fidelity questionnaire which assessed the intensity of any pain, discomfort, or irritation experienced during and after the taVNS application (using 0–10 rating scales, with 0 being none and 10 being the worst imaginable), asked about any other side effects experienced, and asked whether they believe that they received active or sham taVNS and to rate their certainty (0–10, with 0 being extremely unsure and 10 being extremely sure). The third visit involved the same assessment battery, administered once, followed by the EXPECT questionnaire and a semi-structured exit interview.

**Figure 2:**
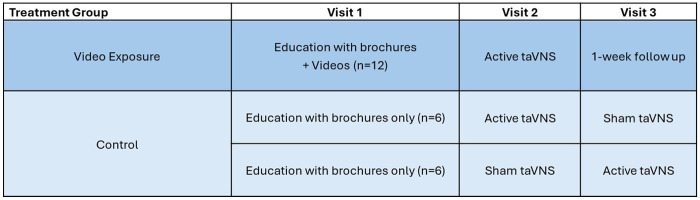
Pilot sham-controlled RCT study design. taVNS, transcutaneous auricular vagus nerve stimulation.

Participants in the control group were considered naïve to the exact location on the ear for active taVNS and thus received a cross-over application of active and sham taVNS conditions in random order for visits 2 and 3 (Figure 2). The active condition was the same as described for the video exposure group. The sham condition used the exact same parameters as the active condition, but the electrodes were placed on the ear lobe, instead of the cymba conchae, which is not innervated by the auricular branch of the vagus nerve (51). The earlobe is a commonly used site for sham taVNS (52, 53). Although setting up the electrodes and applying zero current is the most commonly used sham protocol for taVNS, there are concerns about the effectiveness of blinding with this method (52). The same assessment battery was administered pre and post active and sham taVNS conditions, and the participants in the control group received the tolerability and sham fidelity questionnaire after completing the post-taVNS assessment battery at both visits 2 and 3. The EXPECT questionnaire and a semi-structured exit interview were administered at the end of the 3rd visit.

### Interviews and qualitative analysis

The exit interviews gathered data related to feasibility of conducting taVNS research in this population and focused on the participants' experiences in this study.

A generic qualitative research design, from an interpretivist paradigm, was employed. To enhance to participant comfort and trust, interviews were conducted with racial/ethnic concordant investigators in the preferred language (English or Spanish) for each participant. The interviews were audio recorded and transcribed, and non-English recordings were translated into English for analyses using rapid qualitative analysis (54). Rapid qualitative analysis is widely used for implementation projects such as this, when the goal is to create change in response to the findings rather than to generate new theories. Our goal was to use the interviews to gain insight into participants' experience in the trial in order to better address potential barriers in future studies.

Rapid qualitative analysis was systematically applied in this study according to established protocols (54). The interview guide was used to create structured templates and matrix displays to facilitate data condensation, synthesis, and theme development. Templates were developed collaboratively by the team (MW and JB) and pilot tested to ensure consistency, usability, and relevance. Once consistency was demonstrated, MW completed summaries of transcripts from sessions with Black participants, and JB completed summaries of transcripts for Hispanic/Latino participants. The summaries were aggregated by MW to populate the matrices that enabled systematic comparison between participants.

Both team members who were engaged in the qualitative analysis were physical therapists and pain-scientists, with an interest in health equity, and with extensive experience working with the target populations in clinical settings. All recordings were transcribed in English, and the racial and ethnic backgrounds of the team members who analyzed the transcripts were Black Caribbean (MW, male) and Hispanic/Latino Central American (JB, female).

### Quantitative analyses

Descriptive statistics were used to characterize the participants and their responses for the survey study (i.e., means, standard deviations, frequencies, and percentages). In the pilot RCT, descriptive statistics were used to characterize participants, feasibility outcomes (i.e., recruitment rate, retention, tolerability, and sham fidelity), and HRV. Baseline differences between treatment groups were assessed using Mann-Whitney *U* tests and Chi-Square tests, and the fidelity of the sham was assessed using Chi-Square tests. All statistical analyses were conducted using Statistical Package for the Social Sciences (SPSS) v29 (IBM Corp., Armonk, NY).

## Results

### Survey study

**Figure 3:**
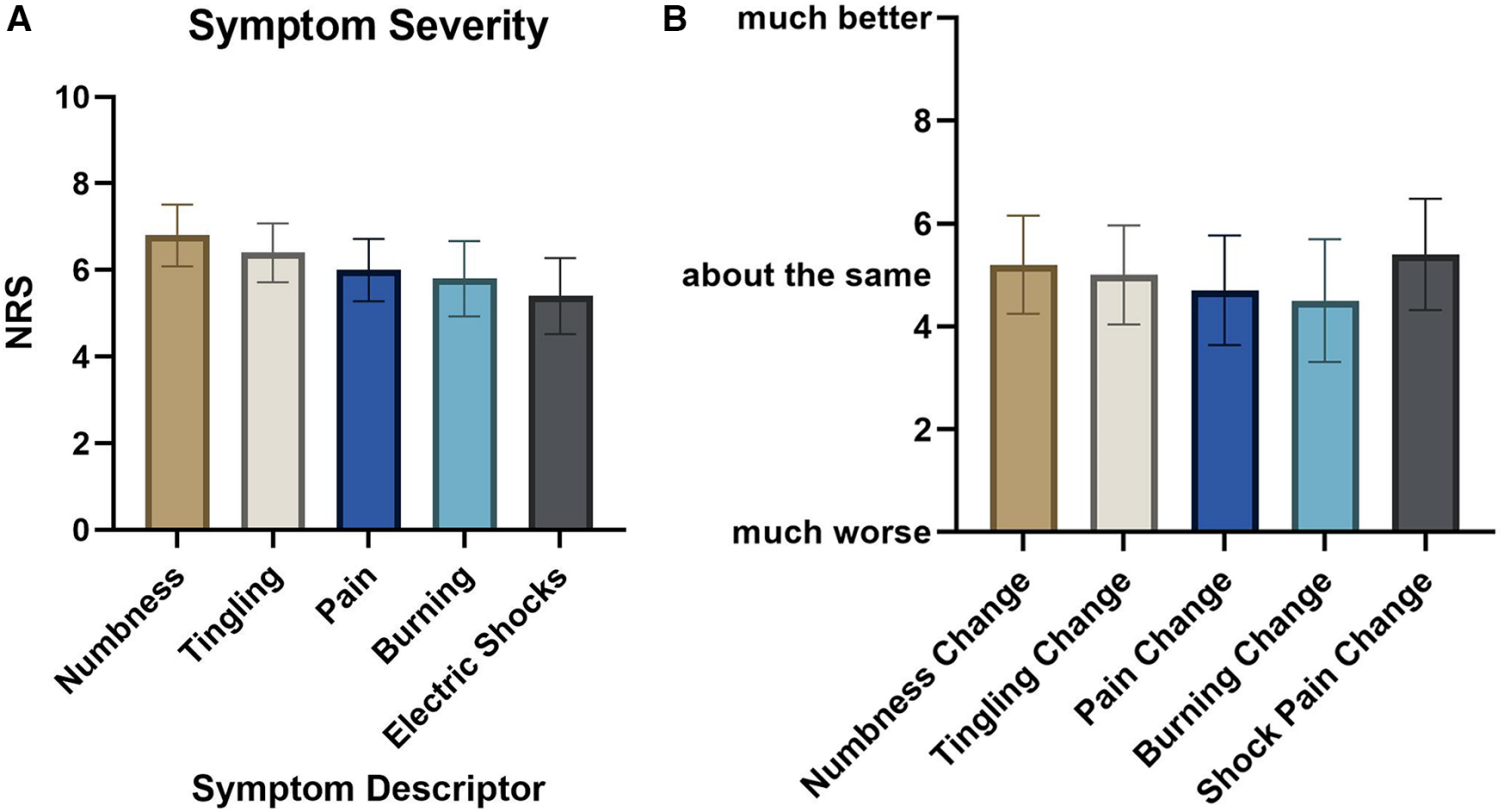
Respondent somatic symptom burden. **(A)** Average symptom severity within the last week. **(B)** Symptom change since ending chemotherapy.

**Figure 4:**
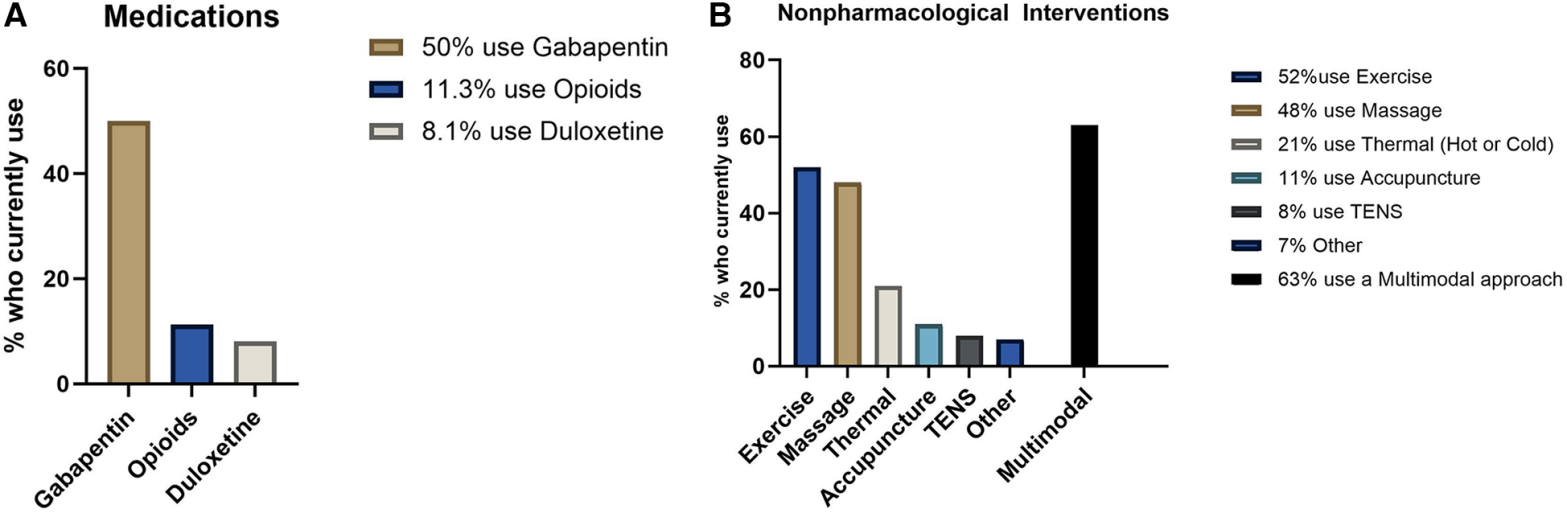
Current treatments used for symptom management. **(A)** Pain medication usage. **(B)** Non-pharmacological interventions. TENS, transcutaneous electrical nerve stimulation.

**Figure 5:**
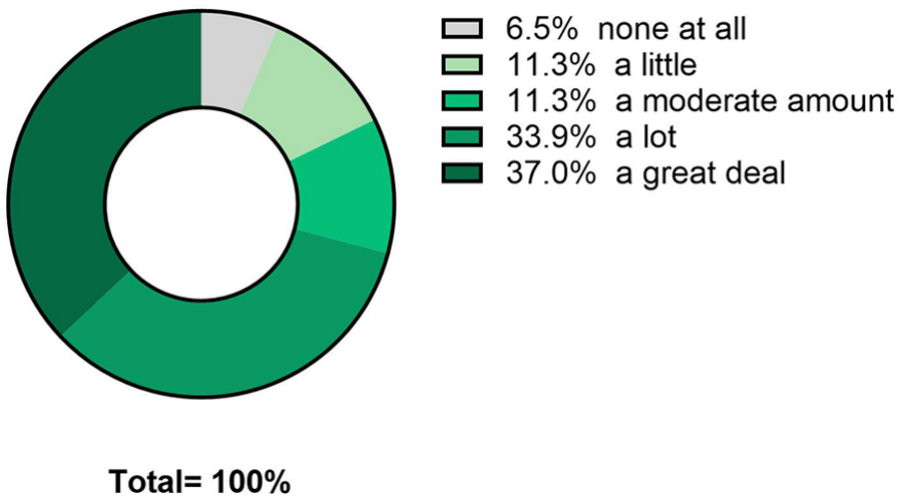
Interest in participating in taVNS research.

Sixty-eight people self-enrolled by initiating the online survey and confirming that they had neuropathy resulting from chemotherapy. Six were removed for completing less than half the survey, and 7 did not complete demographic data but were maintained in the analyses. Thus, there were a total of 62 respondents, including 55 with fully completed data. Summary demographics for the respondents are available in Table 1. Seventy-eight percent of respondents identified as minority racial or ethnic status, and 60% identified as female. Most participants had longstanding CIPN symptoms, with 66% having symptoms for over 1 year. Participants reported high symptom burden (Figure 3A), despite high usage of pharmacologic and nonpharmacologic interventions (Figure 4A,B). On average, there had been no significant reduction of symptom severity after the completion of chemotherapy (Figure 3B). Importantly, there was high interest in taVNS research, with over 82% expressing at least moderate interest in participating (Figure 5). Respondents with high interest in taVNS research expressed a desire to try nonpharmacological interventions, a willingness to “try anything,” or belief that taVNS may target the underlying cause of their symptoms. Those with low interest in participating in taVNS research consistently expressed uncertainty regarding the intervention and its mechanisms of action. Exemplars from the data supporting high and low interest are provided below.

**Table 1:** Survey study respondent characteristics.

Age (mean and SD)	58.5 (10.9)
Gender (% female)	60
Race (%)	
White	67
Black	22
Other	11
Ethnicity (% Hispanic/Latino)	56
Chronicity of CIPN symptoms (%)	
Less than 3 months	13
3–6 months	8
6 months to a year	13
Longer than 1 year	66

Respondents with high interest in taVNS:

“Cause it would be a different alternative than meditation or opioids.” (Respondent 15)

“I’ll try anything safe.” (Respondent 43)

“I have always believed chemo damaged my vagus nerve as I still suffer from gastroparesis and digestive motility issues all with CIPN.” (Respondent 41)

“It will activate the nervous system.” (Respondent 22)

Respondents with low interest in taVNS:

“I don’t understand how the mechanism of stimulating that nerve would help.” (Respondent 1)

“I don’t know what it is.” (Respondents 38)

“No estoy segura no se nada al respecto pero me gustaria saber” Translated: “I’m not sure, I don’t know anything about it, but I’d like to know.” (Respondent 26)

### Pilot RCT

**Figure 6:**
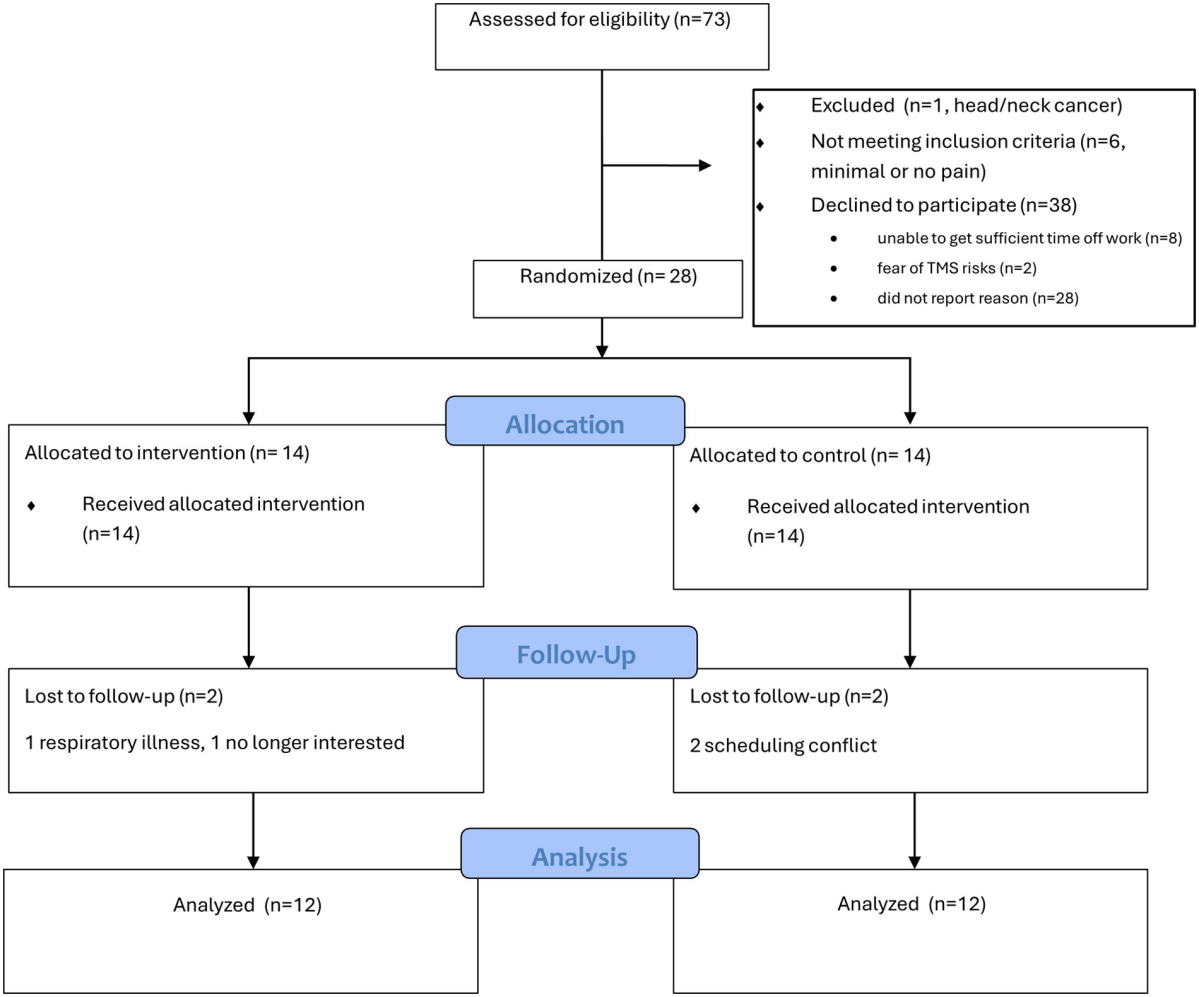
CONSORT flow diagram.

Twenty-eight people were enrolled (17 with CIPN and 11 with DN). Twenty-four completed the trial (Figure 6), with 2 dropping from each of the treatment (video exposure) and control groups. Participant and group characteristics are available in Table 2. Forty-two percent of eligible patients enrolled (28 out of 66), and 86% (*n* = 24) of those enrolled completed the trial. There were no significant differences between groups (*p* > 0.05) for demographic, medical history, or symptom-related factors. Most participants had long-standing symptoms (75% with neuropathy for over 1 year). In addition, average reported symptom levels were moderate to severe (means ranging from 6.1 to 8.0 on a scale of 0–10 for all symptom descriptors), despite widespread use of pain medication (68%). Only 2 of the participants had previously used transcutaneous electrical nerve stimulation (TENS) for pain management.

**Table 2:** Pilot RCT participant characteristics.

	Video (*n* = 14)	Control (*n* = 14)	Total (*n* = 28)	*p* value
Age	58.2 (11.3)	59.0 (7.5)	58.6 (9.4)	0.73
Gender (%Female)	64	79	71	0.40
Race (% Black)	50	64	57	0.51
Ethnicity (% Hispanic)	71	57	64	0.43
Condition (% CIPN)	50	71	61	0.25
Chronicity (% longer than 1 year)	71	79	75	0.50
Using pain medication (% Yes)	64	71	68	0.69
Duloxetine (*n*)	1	0	1	0.31
Gabapentin (*n*)	8	9	17	0.70
Opioids (*n*)	0	1	1	0.31
Experience with TENS (*n*)	0	2	2	0.13
Symptom assessment				
Pain	7.6 (1.8)	6.9 (2.5)	7.2 (2.1)	0.31
Numbness	8.3 (1.8)	7.7 (1.7)	8.0 (1.8)	0.38
Tingling	7.7 (1.9)	7.6 (1.8)	7.7 (1.8)	0.91
Burning	5.6 (4.0)	6.6 (2.4)	6.1 (3.3)	0.76
Shooting pain/Electric shocks	6.1 (3.6)	6.6 (2.9)	6.4 (3.2)	0.84

#### Group differences in expectations for pain relief with taVNS

There was no meaningful difference in the median EXPECT scores between the video and control groups, with respective median values of 8.3 and 8.4, and 95% confidence intervals for the means of 7.3–8.8 and 6.4–8.8. However, the video-exposed group had less variability in EXPECT scores, with a standard deviation of 1.3 compared to 2.1 for the control group.

#### Fidelity of the sham condition

All participants correctly identified the active condition. For the sham condition, 8 thought it was active, 3 were unsure, and only 1 thought that it was sham. Chi-Square Test results were not significant (*p* = 0.08), indicating that the sham is effective even in a crossover design in which participants receive both active and sham.

#### Tolerability

taVNS was well tolerated, with all participants reporting no to moderate tingling irritation during active taVNS, and 1 person reporting high irritation during sham taVNS.

#### Symptom change with taVNS

All symptoms decreased post active and sham taVNS, with greater mean change for the active condition, but the difference was not statistically significant between conditions (*p* values >0.11 across all symptoms; Cohen's *d* values for tingling, numbness, and pain of 0.49, 0.28, and 0.2, respectively). Of the symptoms assessed, the greatest reduction in symptom intensity (mean/median values of active and sham) was noted for tingling (−2.5/−2/0 and −1.0/−1.0), followed by numbness (−2.2/−2.0 and −1.0/−0.5), and then pain (−2.0/−1.5 and −1.5/−0.5).

#### HRV response with taVNS

**Figure 7:**
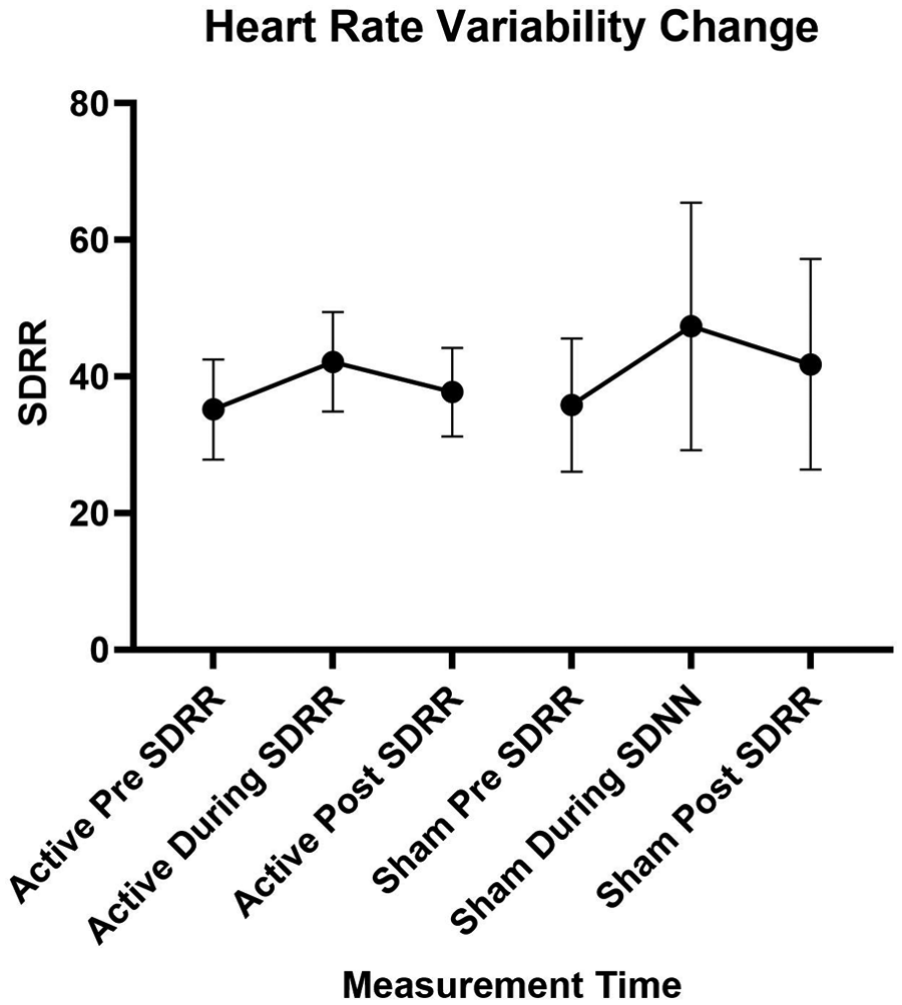
Heart rate variability change. SDRR, standard deviation of the RR interval.

Increased SDRR was noted during the active taVNS with median values increasing from 34.9 (CI = 21.6–44.8) at baseline to 44.8 (CI = 26.5–50.3) during intervention. However, the effect was diminished with post measurements which had a median value of 38.2 (CI = 31.9–45.8). A similar trend was noted for sham stimulation with median values of 30.9 (CI = 19.6–46.2) at baseline, 49.4 (CI = 20.0–64.5) during, and 41.9 (CI = 19.6–56.1) post intervention; however, much greater variability was noted with sham than with active stimulation (Figure 7) (Cohen's *d* of 0.44, *p* = 0.28 for between group differences). Additionally, there was no difference between those with CIPN and DN: both groups had a median increase in SDRR of 4.7 from pre to post active taVNS.

#### Exit interviews

Thirteen of the participants reported receiving some symptom relief from the trial, and 11 did not notice any difference or were unsure. Nevertheless, they all reported overall positive impressions of the experience. Key themes relevant to their overall experience included (1) that it was comfortable, calming, and/or peaceful, (2) that it was interesting and novel, and (3) that it gave them hope.

“Peaceful… It’s hard to find words to describe, kind of nothingness.” (Participant 25)

“it was something I wasn’t accustomed to, felt weird.” (Participant 11)

“It was different…But it was okay. It didn’t hurt….It gave me hope.” (Participant 6)

For those that experienced some symptom relief with the taVNS trial, it was usually partial relief, noticed most often that night or the next morning, and lasted less than 48 h.

Most participants (*n* = 16) stated that they did not perceive any potential barriers for others in their community to participate in this type of research. Among those who did identify barriers, the recurring themes were (1) the length of the protocol (sitting for 3–4 h was difficult for some) and (2) distrust and fear of medical research. To enhance community engagement with this research, participants recommended using social media and conducting research activities in community centers rather than making participants come to our laboratory facility.

## Discussion

The findings from this research support the need and interest for investigating taVNS as a novel treatment for CIPN. In our survey study of 62 people with CIPN, we found high symptom burden despite high usage of pharmacological and non-pharmacological interventions, thus demonstrating a need for new approaches. The current evidence base for CIPN treatments in particular is limited, and therefore the approaches used are based mainly on evidence from other neuropathic pain conditions (55). Duloxetine is the only guideline-recommended pharmaceutical intervention (14), but it may have an unfavorable risk/benefit ratio for many patients. Several recent case studies and case series on invasive spinal cord and dorsal root ganglion stimulation for CIPN report promising results (15). However, these surgical procedures have risks for serious procedural-related complications, such as dural puncture, and biological complications, such as infection and hematoma (16). Further, currently available non-invasive and non-pharmacological treatments lack sufficient evidence for recommendation in treatment guidelines for CIPN. While there is growing evidence supporting exercise as a treatment for this condition (56), exercise tolerance is often limited due to pain and fatigue, and the effect sizes are small (17). Similarly, interventions like acupuncture and scrambler therapy (a noninvasive electro-analgesia device designed for chronic neuropathic and cancer pain) have mixed results and/or small effect sizes (14). The literature on DN treatments is similar. Guidelines state that complete resolution of symptoms is often not achievable, and all of the first line recommended treatments (i.e., tricyclic antidepressants, gabapentinoids, serotonin-norepinephrine reuptake inhibitors, and sodium channel blockers) may have an unfavorable risk/benefit ratio for many patients.

Moreover, there was high interest in taVNS as a potential treatment for CIPN. Notably, a common theme reported among those participants with low interest taVNS was uncertainty regarding the intervention and its mechanisms of action. Recent research suggests that feelings of uncertainty play a key role in low recruitment of minority participants in clinical research, and that participants experience uncertainty when the quality of information is lacking (57). Therefore, the development of high quality educational tools on taVNS may be important to supporting the engagement of underrepresented communities in taVNS research and eventual acceptance of taVNS as a therapy.

In addition to describing the need and demand for taVNS, this research provides important preliminary evidence that it is feasible to conduct taVNS research with an intensive assessment battery in Black and Hispanic/Latino people with peripheral neuropathy. Recruitment and retention of participants is known to be a primary barrier to the successful completion of clinical studies (58), and Black and Hispanic/Latino people are underrepresented in pain research studies, although these same communities are disproportionately affected by chronic pain (59–63). Our study's culturally sensitive approaches may have improved the acceptance rate for potential participants from underrepresented communities. The recruitment team was comprised of people from the same minority communities, we used racial and ethnic matching of researchers and participants, and our recruitment and educational materials were in both English and Spanish. Our findings show that successful enrollment rates of Black and Hispanic/Latino patients can be achieved with a focus on overcoming cultural barriers as described above.

While the 42% overall enrollment rate observed in the pilot RCT is higher than the ranges commonly described in the literature, we hope to improve on this in future studies by employing key lessons learned. From the survey study it became apparent that active recruitment strategies may be needed to ensure representative enrollment of Black participants. Additionally, we plan to employ culturally sensitive educational videos to help manage the uncertainty described by those with low interest in participating in taVNS research. Regarding the sham condition, we plan to continue use of the earlobe but decrease the stimulation intensity to the participants' perception threshold and decrease the duty cycle to 30% in order to minimize the risk of underestimating the effect estimate while hopefully maintaining fidelity of the sham. Based on the information gained from the exit interviews, we plan to implement strategic breaks during the assessment battery to help with the burden of prolonged sitting, and we are planning for the mixed used of laboratory and community facilities for future studies (i.e., conducting the assessment battery in the laboratory but all enrollment and taVNS intervention procedures in the community).

This research also provided data on the potential effects of taVNS on peripheral neuropathy symptoms and HRV in this community. Fifty-four percent of the participants believed that taVNS improved their symptoms. Further, 77% demonstrated an increase in HRV during active taVNS, and this increase was maintained in the post-assessment in 61% of the participants. When the vagus nerve is stimulated, it releases acetylcholine at the synapses with postganglionic cells in the sinoatrial node and atrioventricular node of the heart, modulating heart rate and heart rate variability (64). Thus, the observed increase in HRV suggests target engagement with taVNS in this sample with similar HRV effects as observed in other populations (65–67).

Although this research has achieved its aims, there are limitations to be explored. By focusing on Black and Hispanic/Latino people with peripheral neuropathy, we gained important information from members of communities who are traditionally underrepresented in clinical research; however, this focus also limits the generalizability of our findings, and future studies are needed with samples that are representative of the general population. Additionally, a potential barrier in future studies was the observed similarity of effects with the active and the sham taVNS protocols used. Because it is not innervated by the vagus nerve, the earlobe is widely used as a sham. However, it is known that stimulation of the earlobe is not physiologically inert. In fact, stimulation of the earlobe is a component of cranial electrotherapy stimulation, an FDA-approved therapeutic strategy for the management of insomnia, depression, and anxiety (51, 68). Moreover, fMRI studies have shown that earlobe stimulation deactivates several regions of the limbic system, similarly to the effects of active taVNS (51). A recent systematic review and meta-analysis on transcutaneous vagus nerve stimulation for chronic pain concluded that an active control may underestimate the effect of taVNS and that protocols should have at least 10 sessions to estimate its real impact (53). Thus, the noted effect of the sham and the single session design of this study impacts our ability to estimate the effect of taVNS on PN. We anticipate that greater differences between active and sham protocols will emerge in longitudinal studies with repeated application. Nevertheless, the similarities in acute response to active and sham taVNS is an important consideration for the expected effect size, and sample size needed, when planning future studies.

In summary, taVNS may be a promising non-pharmacological and non-invasive intervention for people with PN, and we found high interest in this modality among this population in the survey study. Further, in a pilot RCT, we found good recruitment and retention rates, as well as positive exit interviews, which provides evidence of good feasibility for conducting large trials of taVNS. This study also provides preliminary evidence for the effectiveness of taVNS in modulating HRV and neuropathic symptoms in people with PN.

## Data Availability

The raw data supporting the conclusions of this article will be made available by the authors, without undue reservation.
