# Supplementary material for: Feasibility of trancutaneous auricular vagus nerve stimulation in Black and Hispanic/Latino people with peripheral neuropathy
Source: Front Pain Res (Lausanne). 2025 Jan 17;5:1516196. doi: 10.3389/fpain.2024.1516196 (PMC11782131; doi:10.3389/fpain.2024.1516196)
Supplement: Supplementary file 1 [file Datasheet1.pdf]

MIAMI

Default Question Block

Pain Management Strategies in People with Chemotherapy Induced Peripheral Neuropathy: a Survey Study

We are asking you to take part in a research study because we are trying to learn more about pain management strategies used by people with chemotherapy induced peripheral neuropathy (CIPN). You will be asked to complete an online survey about your pain, how you manage your pain, and your opinion on a novel treatment for CIPN pain. Completing the survey will take about 20 minutes. There is no compensation for your participation. You can choose to complete or skip any of the questions. Participation is voluntary. There are no negative consequences if you don't want to take it. If you start the survey, you can always change your mind and stop at any time. Taking part in the study involves no risks. You will not benefit directly from being in this research study. You are not required to provide any information that might identify you, but you may provide your information at the end if you wish to be contacted regarding future studies on this topic If you have any questions or concerns about the research, please feel free to contact Marlon Wong, PT, PhD at mwong2@miami.edu or 305-284-2670. If you have questions regarding your rights as a research participant, contact the University of Miami, Human Subject Research Office at hsro@miami.edu or (305)243-3195. By selecting "Next" below, this means you consent to participate in this research project.

Block 1

Do you have pain, numbness, or tingling (e.g., pins and needles) in your hands or feet that started after taking chemotherapy drugs?

- ○ Yes

- No

Block 2

2. How long have you had this problem or pain, numbness, or tingling?

- less than 3 months
- 3-6 months
- 6 months to a year
- longer than 1 year

On a scale of 0-10, with 10 being the worst, how severe are your symptoms on average?

	0	1	2	3	4	5	6	7	8	9	10
Pain											
Numbness											
Tingling											
Burning											
shooting pain or electric shocks											

4. How do you currently manage this problem? Check all that apply

- Medication
- hot or cold packs
- massage
- accupuncture
- exercise
- electrical stimulation (TENS)
- other

please describe other treatments that you use:

[ ]

Please indicate if you take any of the following medications for your pain. Check all that apply:

- ☐ Duloxetine (examples: Cymbalta, Irenka)
- ☐ Gabapentin (examples: Neurontin, Gralise, Horizant, Neuraptine, SmartRx Gaba-V Kit)
- ☐ Opioids (examples: OxyContin, Vicodin, Percocet, Norco, Tramadol)
- ☐ other

Please list any other types of medication that you take for these symptoms specifically:

[ ]

How interested are you in trying treatments other than medication (e.g., electrical stimulation, accupuncture, physical therapy) for this problem?

- ○ None at all
- ○ A little
- ○ A moderate amount
- ○ A lot
- ○ A great deal

Vagus Nerve Stimulation, or VNS, is a noninvasive treatment that has been shown to improve mood and diminish pain. It involves placing an electrode on the ear that delivers mild and nonpainful electrical impulses to stimulate the parasympathetic nervous system. Parasympathetic nervous system activity has been shown to be low in people with cancer and in people with chronic pain.

Do you think that *Vagus Nerve Stimulation* might help your symptoms?

- Definitely not
- Probably not
- Might or might not
- Probably yes
- Definitely yes

Why or why not?

How interested would you be interested in participating in a clinical study using *Vagus Nerve Stimulation* to treat pain?

- None at all
- A little
- A moderate amount
- A lot
- A great deal

What type of cancer did you receive chemotherapy for?

What types of chemotherapy did you receive (please list the names of chemotherapy treatments)

How long ago did you complete chemotherapy treatment?

- I am still receiving chemotherapy
- less than 3 months ago
- 3-6 months ago

- 6 months to a year
- over 1 year

Please state how your symptoms changed after stopping chemotherapy treatment:

	much worse				about the same				much better		
	0	1	2	3	4	5	6	7	8	9	10
Pain											
numbness											
tingling											
burning											
shooting pain or electric shocks											

How much difficulty are you having with managing these symptoms now?

- No difficulty. The problem is well managed, and it is not bothersome.
- A little difficulty. The problem is well managed, but it is still bothersome.
- A lot of difficulty. The problem is manageable at times but not at others.
- Severe difficulty. Nothing seems to help.

What is your date of birth (mm/dd/yyyy):

What is your gender?

- Male
- Female
- Non-binary / third gender
- Prefer not to say

Are you Spanish, Hispanic, or Latino or none of these?

- ○ Yes
- ○ None of these

Choose one or more races that you consider yourself to be:

- ☐ White
- ☐ Black or African American
- ☐ American Indian or Alaska Native
- ☐ Asian
- ☐ Native Hawaiian or Pacific Islander
- ☐ Other ______

What is your country of origin?

______

What zip code do you live in?

______

If you would like to be contacted in the future regarding studies on *Vagus Nerve Stimulation* for pain management, please provide your contact information below:

Name	
Telephone number	
Email address	

Powered by Qualtrics
